# Excitement-Induced Cutaneous Bleeding (Haematidrosis-like) in a Dog

**DOI:** 10.3390/vetsci8120327

**Published:** 2021-12-13

**Authors:** Evi I. Sofou, Anna Gavra, Manolis N. Saridomichelakis

**Affiliations:** Clinic of Medicine, Faculty of Veterinary Science, University of Thessaly, Trikalon Str. 224, GR-43132 Karditsa, Greece; annaga996@gmail.com (A.G.); msarido@vet.uth.gr (M.N.S.)

**Keywords:** haematofolliculohidrosis, haematohidrosis, skin, spontaneous, stigmata, sweat, bleeding

## Abstract

A 15-month-old intact female Pitbull was referred because of recurrent, episodic, self-limiting, excitement-induced bleeding from nontraumatised skin. No abnormalities were detected upon physical examination. Subsequently, the dog went for a walk under the direct supervision of one of the authors, became overexcited and, after approximately five minutes, bloody liquid, with a patchy distribution, appeared along the hair shafts of the face and neck. The affected skin was congested, partially blanching on diascopy and bloody liquid was oozing from the follicular openings. Urticaria, dermographism and hypertension were excluded, the complete blood count and coagulation profile were within the reference ranges and an analysis of the bloody exudate confirmed its blood components. The cutaneous bleeding of the dog followed a self-limited course, with no episodes during the last two years. Clinical and laboratory findings and the long-term evolution of this dog bear striking similarities to haematidrosis, a rare human disease of multifactorial aetiology and equivocal pathogenesis.

## 1. Introduction

Haematidrosis in humans is a rare phenomenon, with multiple causes and triggering factors and with an ill-defined pathogenesis that is clinically characterised by episodic, nontraumatic, bloody sweating [1,2,3,4,5]. To the best of our knowledge, a similar phenomenon in animals has not yet been described, with the only exception some anecdotal reports of haematidrosis in horses with infectious anaemia, purpura haemorrhagica and other bleeding disorders [6] and well-documented cases of bovine neonatal pancytopenia [7]. The aim of this study was to present the case of a dog with clinical manifestations and laboratory findings resembling human haematidrosis.

## 2. Case Presentation

A 15-month-old intact female Pitbull terrier dog was referred because of a 3-month history of recurrent episodes of nontraumatic bleeding from the skin on the head and the neck. According to the owner and the referring veterinarian, each episode lasted for approximately two hours. Based on multiple photographs that had been taken by the owner during previous episodes, it became evident that the exact areas of bleeding differed among the episodes. No drugs had been administered when the episodes first appeared, and all of them had a temporal association with excitement (e.g., they occurred immediately after walking on a leash, when playing, during bathing and when the dog was becoming excited because the owner was returning home). Before referral, serology for *Leishmania* spp.-specific antibodies had been performed, and it was negative. Subsequently, prednisolone (Prezolon, Takeda, Greece) had been administered for 11 days at an initial dose of 0.46 mg/kg twice daily, per os, with gradual tapering without an appreciable impact on the frequency or the intensity of the bleeding episodes. Prednisolone administration had been discontinued seven days before referral.

**Figure 1:**
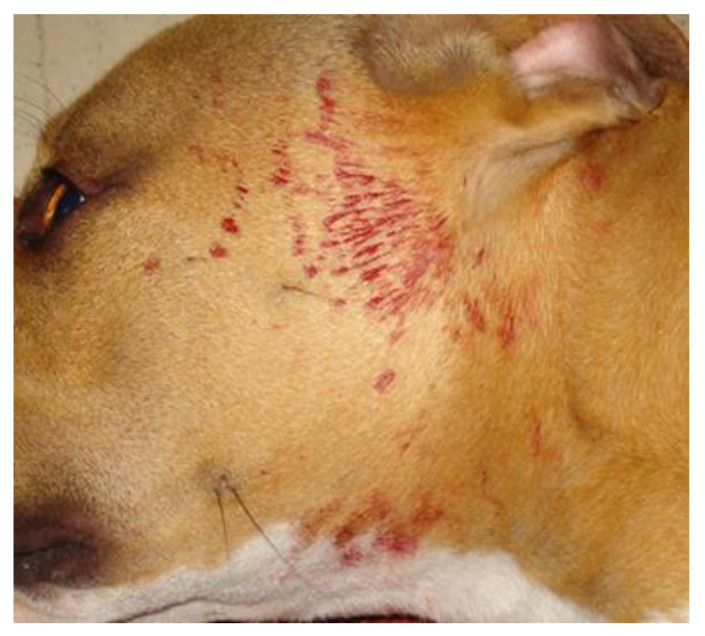
Bloody liquid with a patchy distribution on the hair of the face and the neck of a dog with cutaneous bleeding that occurred after approximately five minutes of overexcitement during walking on a leash.

**Figure 2:**
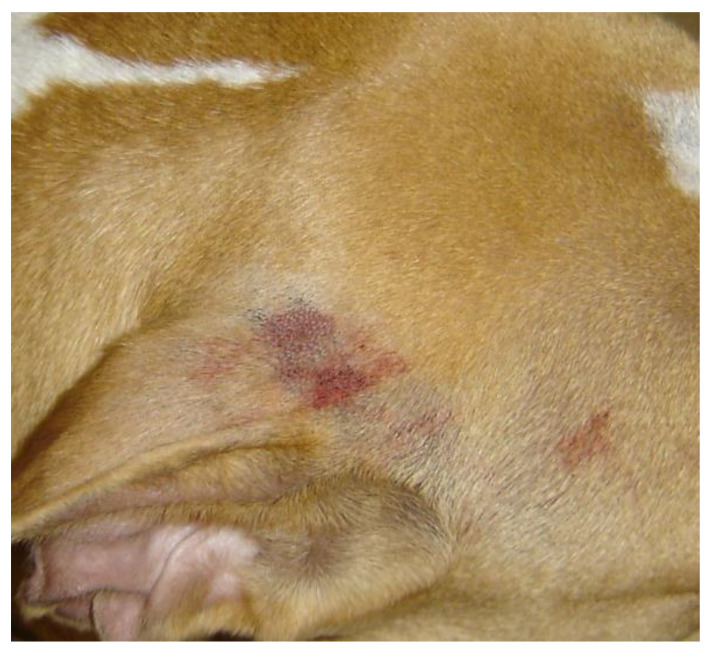
Skin congestion and oozing of bloody liquid from follicular openings in an area of active haemorrhage on the base of the ear pinnae of a dog with cutaneous bleeding. The hair over the affected area has been clipped.

A thorough general physical and dermatologic examination, including palpation of the whole skin, revealed no abnormalities. Subsequently, the dog went for a walk on a leash under the direct supervision of one of the authors. During the walk, the dog became overexcited, but no external trauma or self-trauma occurred. After approximately five minutes, bloody liquid, with a patchy distribution, appeared along some hair shafts of the face and the neck; sinus hairs were not involved (Figure 1). After clipping an affected area, the skin was nonpainful, it was congested and bloody liquid was oozing from the follicular openings (Figure 2). The congested skin partially blanched on diascopy, denoting a combination of haemorrhage and vasodilation. A comparison with the photographs from previous episodes confirmed that the bleeding areas differed from those affected in the past.

Cold-induced urticaria, heat-induced urticaria and dermographism were excluded (no reaction of the skin after application of an ice cube, after application of a heated coin and after manual pressure with a felt-tipped instrument, respectively). Systemic hypertension was also excluded, because the mean systolic blood pressure, measured with the Doppler technique approximately five minutes after the dog returned from the walk, was 130 mmHg. The results of the complete blood count and coagulation profile were within the reference ranges: haematocrit 42.8% (reference values (RF): 37–55%), haemoglobin 14.6 g/dL (RF: 12–18 g/dL), white blood cell count 14,630/μL (RV: 6000–16,900/μL), platelets 427,000/μL (RV: 175,000–500,000/μL), prothrombin time 12 s (RV: <15 s) and activated partial thromboplastin time 14 sec (RV: <15 s). There was only minimal bleeding from the site of a jugular vein puncture. The bloody exudate that was collected with a capillary tube had a microhaematocrit of 38%, whereas the haematocrit of peripheral blood was 42.8%. A Diff-Quik (Merck, Darmstadt, Germany)-stained smear of the exudate that was collected from the follicular openings revealed only normal blood elements.

To exclude histamine-induced and/or catecholamine-induced paroxysmal cutaneous flushing due to a splenic mast cell tumour, carcinoid or pheochromocytoma [8], an abdominal ultrasound was recommended, in addition to skin biopsies from affected and normal skin, serum biochemistry, urinalysis and thoracic radiographs. Due to financial constraints of the owner, the ultrasonographic examination was prioritised and scheduled after a few days. Meanwhile, hydroxyzine (Atarax; UCB, Athens, Greece) at a dose of 2.32 mg/kg twice daily, per os, was prescribed as a precautionary measure for the possibility of histamine-induced anaphylactoid reactions.

None of the recommended examinations were eventually performed, because the episodes ceased two days after consultation. The owner decided to continue hydroxyzine administration for a total of two weeks and then to discontinue the drug. No bleeding episodes were witnessed for the next year, but approximately one year after the consultation, two new episodes occurred, and the owner decided to administer hydroxyzine for two weeks at the same dose as one year ago. At the time of writing, a further 2-year-period has passed without further episodes of cutaneous bleeding or the appearance of any kind of systemic signs, while the dog does not take any medication.

## 3. Discussion

To the best of our knowledge, this is the first case of excitement-induced cutaneous bleeding in a dog. This patient bears striking similarity to emotional stress-induced human haematidrosis.

Diascopy is used to differentiate cutaneous haemorrhage from skin congestion due to severe vasodilation [9]. In the dog of this report, a partial blanching on diascopy was witnessed, which denoted that both vasodilation and haemorrhage were present in the affected skin. In addition, the microhaematocrit and the microscopic examination of the exudate that was collected from the follicular openings confirmed that, in addition to the haemorrhage in the dermis, blood had entered the follicular canals.

Urticaria is characterised by massive vasodilation and plasma extravasation, leading to the appearance of erythematous wheals and, rarely, of haemorrhagic macules [10,11]. Although the dog of this report did not present wheals, it was considered prudent to investigate some causes of urticaria that can be easily confirmed (i.e., cold, heat and dermographism) as possible explanations for the vasodilation. After the exclusion of the above, the diagnostic considerations for the episodic vasodilation included the known causes of paroxysmal cutaneous flushing (mast cell tumours, carcinoids and pheochromocytomas) [8]. Mast cell tumours may originate from internal organs and, most commonly, from the gastrointestinal tract, the spleen and the liver [12]. For this reason, and while waiting for the results of the abdominal ultrasonographic examination, hydroxyzine was prescribed. Pheochromocytoma was considered less likely due to the normal blood pressure soon after the episode. However, pheochromocytoma could not be completely ruled-out, because, in some dogs with this neoplasm, the arterial blood pressure fluctuates within and above the normal range [13]. Even though abdominal imaging was not eventually performed, the self-limiting course of the disease, with no relapses over the last 2 years, along with the absence of systemic signs over a 3-year period, practically excludes all three neoplastic causes of paroxysmal cutaneous flushing.

Nontraumatic cutaneous bleeding in dogs may occur due to clotting abnormalities due to vascular diseases, including vasculitis, congenital and acquired vascular malformations, and due to cutaneous neoplasms, such as haemangiopericytoma, haemangioma, haemangiosarcoma and epitheliotropic T-cell lymphoma [14,15]. Normal platelet counts and a lack of excessive bleeding from the site of a jugular vein puncture make a defect of primary haemostasis unlikely, although a buccal bleeding time and evaluation of platelet function (e.g., through thromboelastography) should, ideally, have been done [16,17]. The normal prothrombin and activated partial thromboplastin times practically ruled out defects of secondary haemostasis as a cause of the cutaneous bleeding [17]. Vasculitis, of various aetiology, was highly unlikely because of the absence of the typical cutaneous lesions [14]. Vascular malformations, such as vascular nevi and arteriovenous fistulas, can be ruled out due to the shifting areas of bleeding during the episodes, the lack of palpable abnormalities of the skin and the self-limiting course of the disease. Finally, bleeding cutaneous neoplasms were not considered valid differentials due to the normal appearance of the skin before the episode and the absence of mass lesions.

Nontraumatic bleeding from follicular openings may occur in deep folliculitis involving sinus hairs [18]. However, the sinus hairs were not bleeding during the episode that occurred in the clinic and did not seem to be involved in the previous episodes (based on the available photographs).

The exclusion of all known diseases that can be considered as possible differentials led to the diagnosis of a haematidrosis-like disease in this dog. Although the strict diagnostic criteria for human haematidrosis have not been defined, the diagnosis of this phenomenon is typically based on a compatible history of episodic, spontaneous cutaneous bleeding, the occurrence of at least one episode under direct medical supervision (in order to exclude nonreported self-trauma), the microscopic confirmation of the bloody nature of the fluid and the exclusion of bleeding disorders [1,2,3,5,19,20,21]. The dog of this report fulfilled all the above diagnostic criteria.

In human haematidrosis, a cutaneous histopathological examination is typically unremarkable and, for this reason, is not usually performed, and it is not considered necessary for a definitive diagnosis [1,4,21,22]. There is only a single case report of a patient where a histopathological examination of the bleeding skin showed blood-filled cavities of unknown aetiology in the dermis [23]. It is unfortunate that, due to the financial constraints and reluctance of the owner, skin biopsies could not be obtained during the bleeding episode witnessed by the authors to allow a comparison of the histopathological appearances between the affected and the nonaffected skin. However, based on the shifting of the bleeding areas among the episodes, the initially grossly and palpably normal skin, the results of diascopy after bleeding and the exclusion of all known differentials, it can be assumed that the histopathological findings would probably have included only vasodilation, congestion and red blood cell extravasation into the dermis and follicular canals, without any structural abnormalities of the skin and its vasculature.

The dog of this report presented many additional clinical similarities to humans with haematidrosis, including a young age at onset, head and neck localisation of the bleeding and a self-limiting course of the disease [1,3,4,20,22], but, also, an important difference: in human haematidrosis, the haemoglobin and/or red blood cell components of the bloody liquid are much lower compared to peripheral blood, because it is mixed with sweat [1,2]. On the contrary, in the dog of this report, the microhaematocrit of the bloody liquid and the haematocrit of the peripheral blood were almost the same. This may be explained by the lower secretory activity of canine compared to human sweat glands.

The reported causes and/or triggers of human haematidrosis include emotional stress, exercise, psychogenic purpura, extragenital menstruation, malaria and idiopathy [1,2,3,4,5,20,21,22,23]. The dog of the present report fits well with the emotional stress-induced human cases, where patients present haematidrosis after excitement [5,20]. The proposed pathomechanism of human emotional stress-induced haematidrosis assumes that, initially, the stress results in an exaggerated activation of the sympathetic nervous system, and this causes extreme peripheral vasoconstriction [5,20,22]. Soon, the initial vasoconstriction is replaced by a reflexive and abrupt vasodilation, microruptures of the walls of the blood vessels and blood extravasation into sweet glands and/or follicular lumens [1,2,3,5,20]. This theory may also explain the response of many of these human patients to anxiolytics and to beta-blockers [1,2,4,5,20,22,23]. The administration of hydroxyzine in our dog for a totally irrelevant reason (safeguard against a severe anaphylactoid reaction if the cause of the clinical signs was a histamine-secreting tumour) may have unintentionally contributed to the ceasing of the episodes due to the known anxiolytic properties of this drug [24]. However, we consider it more likely that the temporal association between hydroxyzine administration and the ceasing of the bleeding episodes was coincidental, and the disease simply followed the typical self-limiting course of human haematidrosis.

## 4. Conclusions

In conclusion, we reported, for the first time in peer-reviewed literature, the case of a dog with episodic, excitement-induced cutaneous bleeding, bearing striking similarity to emotional stress-induced human haematidrosis. If similar cases are encountered in the future with no clinical or laboratory indications of systemic diseases associated with paroxysmal cutaneous flushing, a biopsy of the bleeding and the neighbouring normal-looking skin for histopathological and possibly immunohistochemical examination should be prioritised over imaging or other laboratory examinations to obtain additional information on the location of the bleeding vessels in the dermis; the site of entrance of extravasated red blood cells into the follicular units (i.e., sweat glands, sebaceous glands, infundibulum, isthmus or inferior segment of the hair follicles) and, perhaps, on the possible mechanism of the bleeding.

## Data Availability

Not applicable.
